# Changes of ACE2 in different glucose metabolites and its relationship with COVID-19

**DOI:** 10.1097/MD.0000000000031102

**Published:** 2022-10-14

**Authors:** Yamin Lu, Chenhao Xing, Xiuqin Lv, Cuigai Zhang, Guangxia Liu, Fang Chen, Zhan Hou, Donghui Zhang

**Affiliations:** a Department of Nuclear Medicine, Hebei General Hospital, Shijiazhuang, China; b Hebei North University, Zhangjiakou, China; c Department of Endocrinology, Hebei General Hospital, Shijiazhuang, China; d Physical Examination Center, Hebei General Hospital, Shijiazhuang, China; e Clinical Research Center, Hebei General Hospital, Shijiazhuang, China.

**Keywords:** ACE, ACE2, Ang1-7, AngII, COVID-19, diabetes, prediabetes

## Abstract

To study the changes and effects of angiotensin-converting enzyme 2 (ACE2)/angiotensin 1-7 (Ang1-7) and ACE/AngII in people with different glucose metabolisms and to explore the possible mechanisms underlying the severity of COVID-19 infection in diabetic patients.

**Methods:**

A total of 88 patients with type 2 diabetes, 72 patients with prediabetes (impaired fasting glucose, 30 patients; impaired glucose regulation, 42 patients), and 50 controls were selected. Changes and correlations of ACE2, Ang1-7 and other indicators were detected among the three groups. Patients were divided into four groups according to the course of diabetes: <1 year, 1–5 years, 5–10 years, and >10 years. ACE2 and Ang1-7 levels were compared and analyzed.

**Results:**

ACE2 and Ang1-7 increased with the severity of diabetes (*P*_0_ < .05 or *P* < .01). The levels of ACE2 and Ang1-7 in the longer course group were lower than those in the shorter course group, whereas the levels of ACE, Ang II, and interleukin-6 (IL-6) gradually increased (*P* < .05). Pearson correlation analysis showed that ACE2 was positively correlated with IL-6, FBG, and 2hPBG levels in the prediabetes group. In the diabetic group, ACE2 was positively correlated with Ang1-7 and negatively correlated with ACE, AngII, IL-6, and C-reactive protein levels. Multiple linear regression analysis showed that IL-6 and ACE were the main factors influencing ACE2 in the diabetic group.

**Conclusion subsections:**

ACE2/Ang1-7 and ACE/AngII systems are activated, and inflammatory cytokine release increases in prediabetes. With the prolongation of the disease course, the effect of ACE2/Ang1-7 decreased gradually, while the effect of ACE/AngII increased significantly. Dysfunctions of ACE2/Ang1-7 may be one of the important mechanisms underlying the severity of COVID-19 infection in patients with diabetes.

## 1. Introduction

Angiotensin-converting enzyme 2 (ACE2) is a type I transmembrane glycoprotein composed of 805 amino acids, and its amino acid sequence has 42% homology with ACE.^[1]^ ACE2 is a receptor that antagonizes the RAS system,^[2]^ which is highly expressed in islet cells and the exocrine pancreas, and is closely related to the susceptibility of diabetic patients to COVID-19. The expression and distribution of ACE2 change with age and are affected by many diseases. ACE2 has emerged as a key factor in studies of cellular and molecular mechanisms that may contribute to an increased risk of contracting COVID-19 in individuals with diabetes.^[3]^

It is generally believed that ACE2/angiotensin 1-7 (Ang1-7) and ACE/angiotensin II (AngII) are two important and opposingte functional axes in the RAS system. Under physiological conditions, the two functional axes maintain a dynamic balance and coordinate with each other to maintain body homeostasis. Under pathological conditions, ACE/Ang II can induce oxidative stress, protein glycosylation, inflammation, vasoconstriction, and increased insulin resistance in diabetes mellitus. ACE2/Ang1-7 plays a protective role in beta cell dedifferentiation by improving islet microcirculation and inhibiting islet iNOS mediation.^[4]^

Elevated levels of underlying inflammation are associated with obesity and insulin resistance in type 2 diabetes mellitus (T2DM) patients.^[5]^ T2DM with hyperglycemia is one of the factors leading to elevated ACE2 expression in the lungs and other tissues. ACE2/Ang1-7 and ACE/AngII are bound to have a state of dynamic balance disorder in the development of diabetes, which may be related to the influence of high blood glucose on the systemic inflammatory response and immune system dysfunction.^[6]^

Studies on the changes in ACE2/Ang1-7 during the development of prediabetes and the relationship between ACE2/Ang1-7 and the induction of inflammatory factors are rarely reported at home and abroad. This study aimed to explore the role of ACE2/Ang1-7 in the occurrence and development of diabetes by detecting the levels of ACE2/Ang1-7 in people with different glucose metabolism and different disease courses and their relationship with inflammatory factors to lay a theoretical foundation for further understanding of diabetes and the possible mechanism of the severity of COVID-19.

## 2. Materials and Methods

### 2.1. Study population

According to the diagnostic criteria for Type 2 diabetes in China Guidelines for The Prevention and Treatment of Type 2 Diabetes (2020 Edition),^[7]^ fasting blood glucose (FBG) ≥ 7.0 mmol/L and postprandial blood glucose ≥ 11.1 mmol/L. Prediabetes includes impaired FBG (FBG between 6.1 and 7.0 mmol/L) and impaired postprandial blood glucose regulation (2 h postprandial blood glucose between 7.8 and 11.1 mmol/L). A total of 88 patients (47.5 ± 9.12 years old) with T2DM were enrolled in the outpatient and inpatient groups of Hebei Provincial People’s Hospital from June 2021 to December 2021; 72 patients in the prediabetes group (including 30 patients with impaired fasting glucose and 42 patients with impaired glucose regulation 2 hours after meal) were (45.8 ± 8.94) years old, and 50 patients in the healthy control group were (44.6 ± 8.01) years old. There were no significant differences in sex or age among the three groups.

The exclusion criteria included liver and kidney insufficiency, severe respiratory diseases, tumor radiotherapy and chemotherapy, severe cardiovascular diseases, pregnancy or lactation, severe mental diseases, and infectious diseases. The Ethics Committee of Hebei Provincial People’s Hospital approved informed consent and voluntary participation.

### 2.2. Research method

General information of the patients in the three groups was recorded, including sex, age, diabetes course, height, weight, body mass index, waist circumference, blood pressure, and other indicators. Ang1-7, ACE2, and ACE were detected using ELISA, and AngII was detected using chemiluminescence. Total cholesterol and triglyceride (TG) levels were determined using the GPO-POD method, blood glucose was determined by the hexokinase method, insulin and interleukin-6 (IL-6) were detected by the electrochemiluminescence method, homeostasis model assessment-insulin resistance index (HOMA-IR) reaction insulin resistance level, HOMA-IR = FBG × FINS/22.5, and insulin resistance was determined when the value was >2.8.

### 2.3. Statistical analysis

IBM SPSS Statistics 21.0 (Armonk, NY) software was used for statistical analysis. The measurement data were expressed by mean ± standard deviation (*x* ± *s*), and the normal distribution of variables in each group was tested. One-way ANOVA was used for comparison between groups, Pearson correlation analysis was used for correlation analysis between variables, and multiple linear regression was used to analyze the relationship between dependent variables and independent variables. Statistical significance was set at *P* < .05.

## 3. Results

### 3.1. Characteristics of patients in the study

There were significantly increase of the levels of BMI, waist circumference, systolic blood pressure, total cholesterol, TG, FBG, 2 hours postprandial blood glucose (2hPBG), insulin, and HOMA-IR in the diabetes and prediabetes groups than in the control group (*P* < .05 or *P* < .01). They were higher of TG, FBG, 2hPBG, insulin, and HOMA-IR in the diabetic group than those in the prediabetic group (*P* < .05 or *P* < .01) (Table 1).

**Table 1:** Comparison of general clinical biochemical parameters between control group, diabetic group and prediabetic group (*x* ± *s*).

	Control group (n = 50)	Prediabetic group (n = 72)	Diabetes group (n = 88)
Age (yr)	44.6 ± 8.01	45.8 ± 8.94	47.5 ± 9.12
BMI (kg/m^2^)	23.81 ± 4.56	24.32 ± 4.93	25.37 ± 4.85*
Waist (cm)	86.75 ± 9.88	89.28 ± 12.54*	88.59 ± 10.26*
SBP (mm Hg)	128.48 ± 15.85	134.52 ± 15.59	137.49 ± 13.86*
DBP (mm Hg)	79.17 ± 8.99	78.95 ± 10.24	80.26 ± 12.53
TC (mmol/L)	4.61 ± 0.76	5.45 ± 0.89*	5.56 ± 0.91*
TG (mmol/L)	1.42 ± 0.78	1.81 ± 0.95*	3.39 ± 1.04**▲
LDL-C (mmol/L)	3.32 ± 0.84	3.41 ± 0.89	3.78 ± 0.93
FBG (mmol/L)	4.76 ± 0.53	5.39 ± 0.88*	8.97 ± 1.94**▲▲
2hPBG (mmol/L)	4.89 ± 0.71	8.75 ± 1.20**	14.98 ± 4.03**▲▲
INS (μU/mL)	10.87 ± 3.15	14.61 ± 3.98*	20.56 ± 7.88**▲▲
HOMA-IR	2.29 ± 0.42	3.50 ± 1.05**	8.21 ± 2.01**▲▲

Data presented as mean-standard deviation. *P* < .05 was considered significant.

2hPBG = 2 hour post-prandial blood glucose, BMI = body mass index, DBP = diastolic blood pressure, FBG = fasting blood glucose, HOMA-IR = insulin resistance index, INS = insulin, LDL = low-density lipoprotein, SBP = systolic blood pressure, TC = total cholesterol, TG = triglyceride fatty acid.

Compared with the control group,

* *P* < .05,

** *P* < .01.

Diabetic group compared with prediabetic group,

▲ *P* < .05,

▲

▲*P* < .01.

### 3.2. Comparison of ACE2, Ang1-7 and inflammatory factors among three groups

Compared with the three groups, the levels of ACE2, Ang1-7, ACE, AngII, IL-6, and C-reactive protein (CRP) successively increased, and there were significant differences (*P* < .05, *P* < .01) (Table 2).

**Table 2:** ACE, ACE2, Ang1-7, and IL-6 were compared among control group, diabetic group and prediabetic group (*x* ± *s*).

	Control group (n = 50)	Prediabetic group (n = 72)	Diabetes group (n = 88)
ACE2 (ng/mL)	17.68 ± 3.52	22.05 ± 3.82**	25.52 ± 5.41**▲▲
Ang1-7 (ng/L)	10.18 ± 2.96	11.43 ± 2.92	13.74 ± 3.86**
ACE (ng/mL)	17.73 ± 3.26	24.63 ± 3.87*	30.14 ± 6.01**▲▲
AngII (pg/mL)	35.24 ± 8.56	54.93 ± 11.25**	69.41 ± 16.05**▲▲▲
IL-6 (pg/mL)	3.77 ± 1.38	5.22 ± 1.47*	7.04 ± 1.71**▲▲
CRP (mg/L)	2.36 ± 0.78	3.85 ± 0.95*	5.97 ± 1.68**▲▲

Data presented as mean-standard deviation. *P* < .05 was considered significant.

ACE2 = angiotensin-converting enzyme 2, Ang II = angiotensin II, Ang1-7 = angiotensin 1-7, CRP = C-reactive protein, IL-6 = interleukin-6.

Compared with the control group,

* *P* < .05,

** *P* < .01.

Diabetic group compared with prediabetic group,

▲ *P* < .05,

▲

▲*P* < .01.

### 3.3. Comparison of various indices between different disease courses in the diabetes group (course of disease < 1 year group, course of disease 1–5 years group, course of disease 5–10 years group, course of disease > 10 years group)

**Figure 1.**
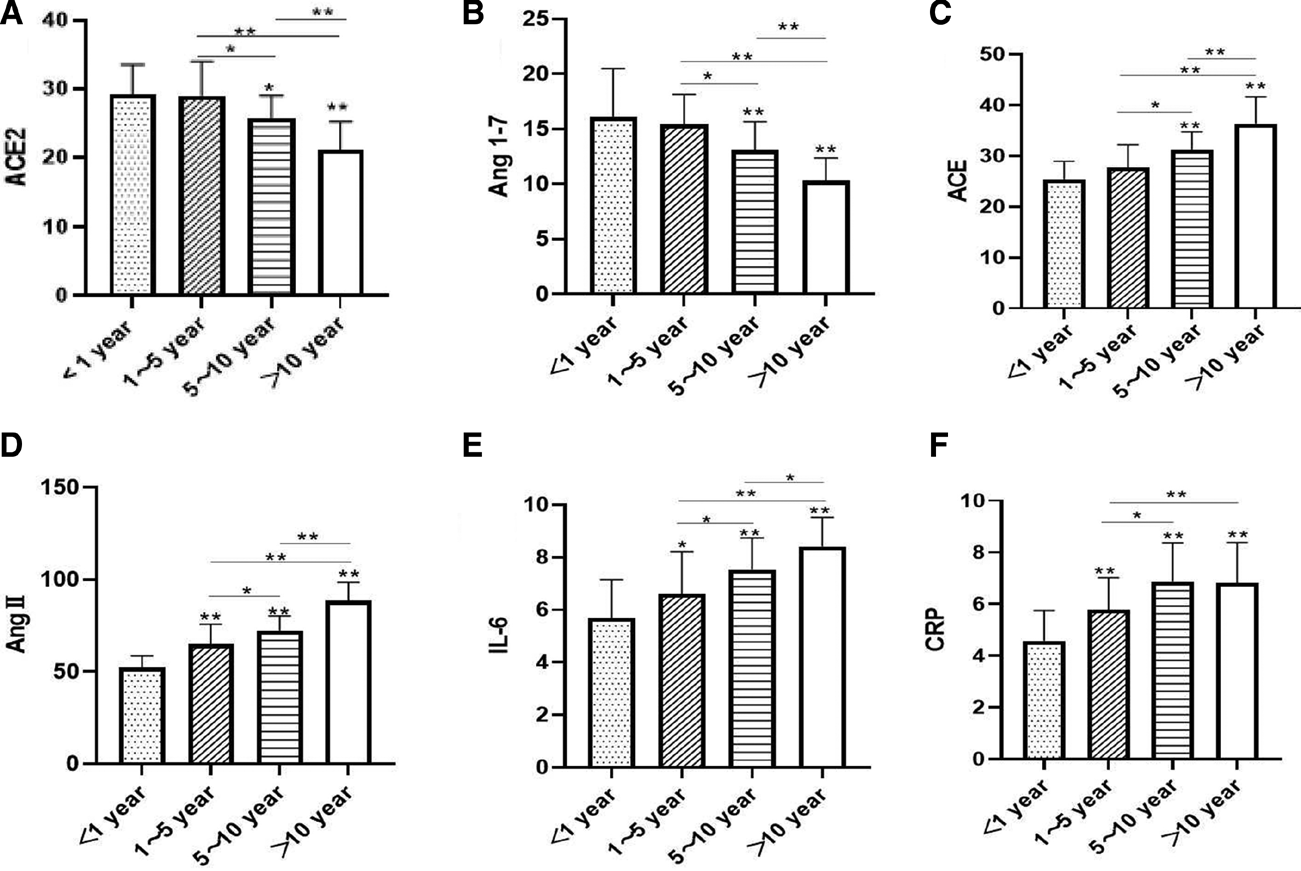
Changes in RAS system indicators and inflammatory factors: compared with the <1 year group and 1 to 5 year group, the levels of ACE2 and Ang1-7 in 5 to 10 year group and >10 year group were significantly decreased, while the levels of FBG, 2hPBG, ACE, AngII, IL-6, and CRP were significantly increased (*P* < .05, *P* < .01). ACE, AngII, and IL-6 increased gradually with prolongation of the disease course, while ACE2 and Ang1-7 decreased gradually with prolongation of the disease course (*P* < .05 or *P* < .01). 2hPBG = 2 hours postprandial blood glucose, ACE2 = angiotensin-converting enzyme 2, Ang II = angiotensin II, Ang1-7 = angiotensin 1-7, CRP = C-reactive protein, FBG = fasting blood glucose, IL-6 = interleukin-6.

Changes in RAS system indicators and inflammatory factors: Compared with the <1 year group and 1 to 5 year group, the levels of ACE2 and Ang1-7 in 5 to 10 year group and >10 year group were significantly decreased, while the levels of FBG, 2hPBG, ACE, AngII, IL-6, and CRP were significantly increased (*P* < .05, *P* < .01). ACE, AngII, and IL-6 increased gradually with prolongation of the disease course, while ACE2 and Ang1-7 decreased gradually with prolongation of the disease course (*P* < .05 or *P* < .01) (Table 3 and Fig. 1A–F).

**Table 3:** Comparison of related indexes of different course of disease in diabetic group (course of disease < 1 year group, course of disease 1–5 years group, course of disease 5–10 years group, course of disease > 10 years group) (*x* ± *s*).

	<1 yr group	1–5 yr group	5–10 yr group	>10 yr group
Proportion of population (%)	27.27 (24/88)	23.86 (21/88)	22.72 (20/88)	26.14 (23/88)
FBG (mmol/L)	8.36 ± 1.55	8.49 ± 1.83	9.22 ± 1.82	9.81 ± 2.15*▲
2hPBG (mmol/L)	13.36 ± 3.82	13.89 ± 4.63	16.09 ± 3.6*	16.71 ± 2.85**▲

Data are presented as number (percentage) or mean ± standard deviation. Statistical significance was set at *P* < .05.

2hPBG = 2 hours postprandial blood glucose, FBG = fasting blood glucose.

Compared with the <1 year group,

* *P* < .05,

** *P* < .01.

Compared with the 1–5 years group,

▲ *P* < .05,

▲

▲*P* < .01.

### 3.4. Correlation analysis

Pearson correlation analysis showed that ACE2 was positively correlated with IL-6, FBG, and 2hPBG in prediabetes group (*R* = 0.271, 0.572, 0.843, *P* < .05 or *P* < .01). There was a positive correlation between ACE2 and Ang1-7 in diabetic group (*R* = 0.391, *P* < .01). It was negatively correlated with ACE, AngII, IL-6, and CRP (*r* = −0.563, −0.497, −0.515, −0.243, *P* < .05 or *P* < .01).

### 3.5. Multivariate linear regression analysis of ACE2 influencing factors in diabetic patients

Multivariate linear regression analysis (FBG, 2hPBG, Ang1-7, ACE, Ang II, IL-6, and CRP) was performed with ACE2 as the dependent variable and seven statistically significant variables between groups with different disease courses as independent variables. The results showed that ACE and IL-6 were the main factors influencing ACE2 (*P* < .01), which together explained 39.9% of the total variation in the regression equation (Table 4).

**Table 4:** Multivariate linear regression analysis of ACE2 influencing factors between different stages of diabetes mellitus (course of disease < 1 year group, course of disease 1–5 years group, course of disease 5–10 years group, course of disease > 10 years group) (n = 88).

	B	SE	β	*t*	*P*	95% CI
(constant)	38.575	4.405		8.756	.000	
ACE	−0.321	0.108	−0.357	−2.963	.004	28.85–31.42
IL6	−0.977	0.312	−0.309	−3.131	.002	6.67–7.40

*R* = 0.669, *R*^2^ = 0.447, adjusted *R*^2^ = 0.399, *F* = 9.239, *P* < .001.

Multivariate linear regression analysis was performed with ACE2 as the dependent variable and FBG, 2hPBG, Ang1-7, ACE, Ang II, IL-6, and CRP as independent variables between groups with different disease courses.

2hPBG = 2 hours postprandial blood glucose, β = standardized partial regression coefficient, ACE2 = angiotensin-converting enzyme 2, Ang II = angiotensin II, Ang1-7 = angiotensin 1-7, B = partial regression coefficient, CI = confidence interval, CRP = C-reactive protein, FBG = fasting blood glucose, IL-6 = interleukin-6, SE = standard error.

## 4. Discussion

T2DM is a common complication in patients with COVID-19 and an important risk factor for its severity, poor prognosis, and increased mortality.^[8]^ However, the mechanism by which T2DM leads to severe COVID-19 remains unclear. The common link between metabolic diseases and coronavirus is an inflammatory response; therefore, we should actively seek ways to control inflammation and reduce the incidence of inflammatory responses and complications.

ACE2 is an extracellular enzyme expressed in different organs of the human body, including the pancreas.^[9]^ It mainly exists in islets and is preferentially expressed in insulin-producing β cells. It is also expressed in exocrine pancreatic microcirculation capillary endothelial cells/pericells and periislet capillaries and is involved in activating RAS circulation in systemic or local tissues.^[10]^ ACE2 can improve the endothelial function of islet microvessels to protect islet function, ACE2/Ang1-7 can regulate glucose homeostasis by regulating GAD67/GABA signal transduction in β cells, improving β-cell function, and delaying the induction of diabetes.^[11]^ ACE2 can reduce oxidative stress and apoptosis in islet cells and improve insulin secretion.^[12]^

The dynamic balance of immune metabolism is destroyed in prediabetic individuals, and the percentage of pro-inflammatory cells is significantly increased by the activation of monocytes and macrophages, while the percentage of anti-inflammatory cells is unchanged.^[13]^ Oxidative stress and increased inflammation are associated with blood glucose levels and glucose intolerance.^[14]^

This experiment also showed that prediabetic people already had dyslipidemia of blood glucose and insulin resistance, and elevated TG was deposited in fat cells, reducing insulin sensitivity.^[15]^ Glucotoxicity caused by blood sugar disorders can cause amyloidosis in the islet cells. The increased release of the inflammatory mediators IL-6 and CRP activates the two functional axes of the RAS system, ACE/Ang II and ACE2/Ang1-7, resulting in corresponding body effects. The adaptive response of ACE2/Ang1-7 antagonizes overactive ACE/Ang II, which is also a mechanism of body self-protection. During progression to the diabetic stage, this type of immune metabolism function abnormality is more obvious. The level of ACE2 in the prediabetic group was higher than that in the control group, which may be due to the high expression of ACE2 in islet tissue, leading to the production of Ang1-7. An in vitro assay showed^[16]^ that with an increase in glucose concentration, ACE2 gene overexpression increased significantly after 72 h. In patients with chronic and/or persistent hyperglycemia, upregulation of ACE2 and its potential glycosylation and dysfunction may be associated with complications observed in patients with COVID-19. Meanwhile, this study showed that ACE2 was positively correlated with IL-6, FBG, and 2hPBG, especially with blood glucose, indicating that in prediabetes, ACE2 expression is mainly regulated by blood glucose. Therefore, glucose control in prediabetes can protect the function of β cells, ACE2, and its receptor Mas and reduce the adverse effects of ACE/Ang II.

If blood glucose is not effectively controlled and develops into diabetes, with the aggravation of glucose and lipid metabolism disorder and insulin resistance, the corresponding cells or organs release more inflammatory mediators.^[17]^ In this study, ACE2 was negatively correlated with ACE, AngII, IL-6, and CRP in the diabetic group, but not with blood glucose. These results indicate that the expression of ACE2 is mainly affected by inflammatory mediators in the diabetes stage (the release of such inflammatory mediators may be largely caused by repeated fluctuations of blood glucose). Therefore, effective control of blood glucose fluctuations and inhibition of inflammatory hyperexpression in the stage of diabetes can weaken the damage caused by ACE/Ang II in β cells. The relatively enhanced activation of ACE2/Ang1-7/MasR reverses and prevents local and systemic dysfunction,^[18]^ thereby improving lipid distribution and insulin resistance by regulating insulin action and reducing inflammation.

As the disease progresses, the dynamic balance between ACE/AngII and ACE2/Ang1-7 is disrupted. In patients, the expression of ACE2 depends on disease progression. In the early stages of diabetes, ACE2 is upregulated and downregulated during the late stages.^[19]^ This experiment showed that compared with the <1 year group and the 1 to 5 year group, the levels of ACE2 and Ang1-7 in the 5 to 10 year group and >10 year group were lower, while the levels of FBG, 2hPBG, ACE, AngII, IL-6, and CRP were higher. ACE, Ang II, and IL-6 increased gradually with the prolongation of the disease course, while ACE2, Ang1-7 decreased gradually with the prolongation of the disease course. This is similar to the results of some studies^[20]^: the plasma ACE2 concentration in patients with chronic diabetes is much lower than that in healthy controls, Patients with diabetes with low plasma ACE2 levels may be susceptible to severe COVID-19 and have a poor prognosis. The possible mechanism of this result is that T2DM is a progressive disease, in which glucolipid toxicity and abnormal immune metabolism caused by inflammatory destruction cause irreversible damage to islets and β cells, leading to a progressive decrease in ACE2 expression. Direct β-cell damage and cytokine-induced insulin resistance can lead to the deterioration of glycemic control in diabetic patients.^[21]^

Studies have shown that decreased ACE2 content leads to increased expression of ACE and angiotensin II type 1 receptor, dysregulation of glucose homeostasis, decreased insulin content, and increased oxidative stress in islet β-cells.^[22]^ In this study, multiple linear regression analysis of the factors affecting ACE2 between the groups with different disease courses showed that ACE and IL-6 were the main factors affecting ACE2, explaining 39.9% of the total variation in the regression equation. It is suggested that in the late stage of diabetes, the self-anti-inflammatory effect is weak and the inflammatory effect is dominant, which may cause “inflammatory factor storm.” This may also explain how T2DM contributes to the severity of COVID-19: the overexpression of ACE2, a functional receptor for COVID-19, also indirectly promotes the entry of the virus into cells and increases the susceptibility of diabetic patients to COVID-19 infection.^[23]^ Later stages of diabetes: ACE2/Ang1-7 expression drops sharply, resulting in massive infection of pancreatic islet cells by viruses, direct damage and depletion of pancreatic β cells, and damage to glucose homeostasis and metabolism of patients.^[24]^ The body is not sufficient to ht the invasion of inflammatory factors and the ACE/Ang II stress response to the organs of the whole body and death.

There are shortcomings in this study: there are new patients and patients taking medication in the diabetic population, which may cause an influence bias of drugs.

## 5. Conclusions

Data in the current study demonstrate that prediabetes increases ACE2/Ang1-7, ACE/AngII system activation, and inflammatory factor release. With prolongation of the disease course, the effect of ACE2/Ang1-7 gradually decreased, and the effect of ACE/AngII and inflammatory factors significantly increased, which accelerated the occurrence and deterioration of diabetes. Dysfunction of ACE2/Ang1-7 may contribute to the severity of COVID-19 in patients with diabetes.

## Author contributions

YL, CX, and DZ designed the study, collected data, analyzed relevant information, wrote the manuscript, and approved the final submission.

**Formal analysis:** Guangxia Liu, Fang Chen, Zhan Hou, Cuigai Zhang.

**Investigation:** Yamin Lu, Xiuqin Lv.

**Methodology:** Chenhao Xing, Yamin Lu.

**Project administration:** Yamin Lu, Chenhao Xing, Xiuqin Lv.

**Writing – original draft:** Yamin Lu, Chenhao Xing.
